# Separation Anxiety and Personality Domains in a Dimensional Perspective: A Cross-Sectional Study in a Sample of Adults with Personality Disorders

**DOI:** 10.62641/aep.v53i2.1811

**Published:** 2025-03-05

**Authors:** Giovanni Genovese, Carmenrita Infortuna, Valentina Clementi, Fiammetta Iannuzzo, Fabrizio Turiaco, Carmela Mento, Maria Rosaria Anna Muscatello, Antonio Bruno, Gianluca Pandolfo

**Affiliations:** ^1^Psychiatry Unit, Polyclinic Hospital University of Messina, 98125 Messina, Italy; ^2^Department of Biomedical and Dental Sciences, Morphological and Functional Images, University of Messina, 98125 Messina, Italy

**Keywords:** separation anxiety, personality disorders, personality traits, dimensional approach

## Abstract

**Background:**

Recent developments have highlighted the importance of separation anxiety across the lifespan, positioning it as a longitudinal psychopathological dimension. Few studies in the past decade, have explored this correlation within the context of other psychiatric disorders. This study aims to assess the presence of childhood and adulthood separation anxiety in a sample of adults with personality disorders, and its potential contribution to specific personality domains.

**Methods:**

A sample of 102 patients (39% male, 61% female) with a principal diagnosis of “Unspecified Personality Disorders” according to the Diagnostic and Statistical Manual of Mental Disorders – 5° edition – text revision (DSM-5-TR) was recruited. The patients were assessed using the following instruments: the Structured Clinical Interview for Separation Anxiety Symptoms and the Personality Inventory for DSM-5-Brief Form (PID-5-BF). Correlation and linear regression analyses were performed.

**Results:**

Both childhood and adulthood separation anxiety were positively correlated with all PID-5 domains except “Antagonism” (*p* = 0.352/0.067). The linear regression analysis showed that only adult separation anxiety was a direct predictor of the personality domains “Negative Affectivity” (*p* = 0.002), “Detachment” (*p* = 0.008), and “Psychoticism” (*p* = 0.028).

**Conclusions:**

Our study is the first to highlight the potentially crucial role of adult separation anxiety levels in personality disorders. Unexpectedly, childhood separation anxiety did not predict personality domains. The presence of separation anxiety should be considered a potential developmental obstacle to a healthy transition toward a well-rounded adult personality organization.

## Introduction

Separation anxiety disorder (SAD) is characterized by excessive and inappropriate fear or anxiety regarding separation from home or attachment figures [1]. While separation anxiety is considered a normal aspect of childhood development, when it becomes excessive in frequency or intensity, it can hinder emotional regulation and impair interpersonal functioning. Rooted in psychoanalytic object relations and attachment theories, separation anxiety has significant biological underpinnings and is considered a biologically related anxiety disorder [2]. Twin studies have shown heritability estimates ranging from 0.21 to 0.74, with higher heritability in females (52%) compared to males (26%) [3]. Additionally, biological vulnerability is supported by evidence of hypersensitivity to carbon dioxide (CO_2_) [4] which is thought to be an endophenotype shared among SAD, panic disorder (PD), and anxiety sensitivity [5].

The continuity spectrum between SAD and PD has been corroborated by research showing that childhood SAD increases the likelihood of developing PD later in life [6]. Given the importance of separation anxiety during development, it is not surprising that SAD has a lifetime prevalence of 6.6% in the general population [7], with clinical samples showing rates as high as 42% [8]. SAD often co-occurs with other psychiatric disorders, including PD, other anxiety disorders [4, 9, 10], obsessive-compulsive disorder [11, 12], and depression [13].

Recently, SAD’s relevance across the lifespan has led to its inclusion in diagnostic criteria for both adolescents and adults in the Diagnostic and Statistical Manual of Mental Disorders – 5° edition (DSM-5).

A key area of interest in SAD research is its relationship with personality disorders. Personality disorders are often preceded by early-life trauma, and it remains unclear whether SAD is a risk factor for the development of maladaptive personality traits or whether they share common vulnerabilities [14]. Few studies in the past decade evaluated the correlation of personality disorder with SAD, mainly in the context of other psychiatric disorders. In a sample of patients with social anxiety disorder, SAD score was associated with current depression, suicide ideations, and avoidant personality disorder [15]. Among patients with obsessive-compulsive disorder (OCD), high dependency and lower extraversion traits are associated with SAD [16]. Interestingly, patients with both childhood and adult SAD showed higher rates of Cluster C and B personality disorders compared to other patients [17]; individuals with Cluster C traits tend to be characterized by anxiety or fearfulness, while Cluster B traits are marked by emotional instability and dramatization, particularly in relational contexts where fears of abandonment or rejection are prevalent.

The authors stress the importance of distinguishing separation anxiety from these manifestations, noting that people with adult SAD are primarily concerned about the safety of attachment figures, differently from those with personality disorders who focus on their inability to cope with loss.

The alternative model for personality disorders proposed in the DSM-5 offers a dimensional approach to diagnosis, providing a more detailed understanding of personality traits. This framework attempts to overcome several limitations of the categorical approach, such as the frequent overlap among criteria that leads to an overuse of “Personality Disorder Not Otherwise Specified” (PD-NOS) diagnosis; on the other hand, the Diagnostic and Statistical Manual of Mental Disorders – 5° edition – text revision (DSM 5 TR) states that “other specified or unspecified personality disorder is often the correct (but mostly uninformative) diagnosis” [1]. Given the prevalence of unspecified personality disorders, investigating separation anxiety in this sample might yield results more in line with the real world; based on these premises, this study aims to evaluate the presence of childhood and adulthood separation anxiety in a sample of adults diagnosed with other specified or unspecified personality disorder, and its possible contribution to specific personality domains. We hypothesize that levels of separation anxiety experienced as a child or adult are associated with maladaptive personality traits, particularly in the domain of Negative Affectivity.

## Materials and Methods

### Subjects

Participants were recruited using a convenience sampling method. Both inpatients and outpatients, aged between 18 and 70 years, who were referred to the Psychiatry Unit of the University Hospital “G. Martino” of Messina and the Psychiatric Residential Clinic “Colle Cesarano” of Tivoli (RM) between January and December 2023, were consecutively included in the study. All participants had a principal diagnosis of “Unspecified Personality Disorders” according to the DSM-5-TR [1]. Exclusion criteria at the time of enrollment included any conditions that could potentially interfere with the study’s outcomes, such as significant medical comorbidities, organic brain disorders, neurocognitive disorders, or current alcohol or substance addiction. Participants were informed about the study’s objectives, methods, and potential benefits. Data were collected anonymously, no incentives were provided for participation, and individuals had the option to withdraw from the study at any time. The research protocol followed the guidelines of the Declaration of Helsinki and was approved by the Ethics Committee of the University of Messina (Prot. N°69/16 - 01/07/2016). All participants provided written informed consent before taking part in the study.

### Measures

To assess both past and current experiences of separation anxiety, as well as specific personality traits, the following instruments were used:

• The Structured Clinical Interview for Separation Anxiety Symptoms (SCI-SAS) [18] evaluates separation anxiety in adults, distinguishing between childhood and adulthood symptoms. Responses are recorded on a four-point scale: 0 (not at all), 1 (sometimes), 2 (often), or ? (I do not recall). Scores from the eight items on both the SCI-SAS-C (childhood) and SCI-SAS-A (adulthood) were summed to generate continuous measures of separation anxiety symptoms in childhood and adulthood frames (range for each scale: 0–16). The SCI-SAS demonstrates good internal consistency, with Cronbach’s alpha values of 0.79 for the childhood scale and 0.85 for the adult scale.

• The Personality Inventory for DSM-5-Brief Form (PID-5-BF) [19] is a 25-item self-report questionnaire assessing five broad personality domains: Negative Affectivity, Detachment, Antagonism, Disinhibition, and Psychoticism. Each domain represents distinct patterns of behavior, emotions, and interpersonal functioning. Responses are recorded on a four-point Likert scale (ranging from 0 = very false or often false to 3 = very true or often true). The longitudinal stability of PID-5 traits has been well-established [20].

### Statistical Analysis

Only fully completed questionnaires were analyzed. The Shapiro-Wilk test has been performed, then data were analyzed with descriptive statistics (Mean ± standard deviation (SD), Median (P25, P75), or numbers and percentages, as requested). A correlation analysis was performed to evaluate possible associations between personality traits and levels of children and adult separation anxiety. A linear regression analysis, in which the PID-5 domains were considered dependent variables and SCI-SAS constructs were included in the equation, was performed to assess what kind of separation anxiety could play the role of specific predictor towards the different traits of personality. Data were analyzed with IBM SPSS Statistics 23.0 (IBM Corp, Armonk, NY, USA). A *p*-value of <0.05 was considered statistically significant.

## Results

### Descriptive Features

The final sample consisted of 102 patients, 61% of whom were female (n = 62), with ages ranging from 18 to 66 years (mean age ± SD = 41 ± 12.8 years). Table 1 presents the demographic and clinical characteristics of the study sample. Regarding the SCI-SAS, low levels of separation anxiety were observed both during childhood (Median (P25, P75) = 6 (2, 9)) and adulthood (Median (P25, P75) = 7 (4, 10)). In terms of the PID-5, higher scores were recorded in the “Negative Affectivity” domain (Median (P25, P75) = 1.8 (1.2, 2.2)), followed by the “Detachment” (Median (P25, P75) = 1.4 (0.8, 1.8)) and “Disinhibition” (Median (P25, P75) = 1.4 (1, 1.8)) domains.

**Table 1.** **Descriptive statistics: demographic and clinical characteristics of the participants (N = 102)**.

		Value [Mean ± SD / Median (P25, P75) / n (%)]	
Age		41 ± 12.8	
Gender			
	Male	40	39%
	Female	62	61%
Relationship status			
	Single	55	53.9%
	Married	28	27.5%
	Cohabitant	6	5.9%
	Widower	3	2.9%
	Divorced	10	9.8%
Education			
	Primary school	5	4.9%
	Secondary school	41	40.2%
	College	38	37.3%
	Degree	15	14.7%
	postgraduate	3	2.9%
Working conditions			
	Worker	6	5.9%
	Unemployed	41	40.2%
	Housewife	7	6.9%
	Student	8	7.8%
	Other	40	39.2%
SCI-SAS			
	SCI-SAS child	6 (2, 9) (Range 0–16)	
	SCI-SAS adult	7 (4, 10) (Range 0–16)	
PID-5			
	Negative Affectivity	1.8 (1.2, 2.2) (Range 0–3)	
	Detachment	1.4 (0.8, 1.8) (Range 0–3)	
	Antagonism	0.6 (0.2, 1.2) (Range 0–3)	
	Disinhibition	1.4 (1, 1.8) (Range 0–3)	
	Psychoticism	1.2 (0.6, 1.8) (Range 0–3)	

SCI-SAS, Structured Clinical Interview for Separation Anxiety Symptoms; PID-5, Personality Inventory for DSM-5; SD, standard deviation.

### Correlation and Regression Analysis

Table 2 presents the results of the Spearman correlation analyses conducted to assess potential associations between personality traits and separation anxiety levels. The analysis revealed that both childhood and adulthood separation anxiety were positively correlated with all PID-5 domains, except for “Antagonism” (*p* = 0.352/0.067).

**Table 2.** **Correlation analysis (r values) between personality domains, SCI-SAS-C (childhood), and SCI-SAS-A (adulthood)**.

	Negative Affectivity	Detachment	Antagonism	Disinhibition	Psychoticism
SCISAS child	0.388***	0.200*	0.094	0.200*	0.286**
SCISAS adult	0.477***	0.343***	0.182	0.256**	0.356***

* *p* < 0.05; ** *p* < 0.01; *** *p* < 0.0001.

Table 3 presents the linear regression analysis conducted to assess the potential predictive role of the two SCI-SAS constructs (as independent variables) on the five PID-5 domains (as dependent variables). The results indicate that only adult separation anxiety was a direct predictor of the PID-5 domains “Negative Affectivity” (*p* = 0.002), “Detachment” (*p* = 0.008), and “Psychoticism” (*p* = 0.028). Childhood separation anxiety did not significantly contribute to the prediction of personality traits.

**Table 3.** **Linear regression analysis**.

		Unstandardized coefficients		Standardized coefficients		
Dependent variable	Predictors	B	S.E.	Beta	*t*	*p*
Negative Affectivity ^a^ (Model 1)	(Constant)	1.142	0.128		8.887	<0.0001
	SCISAS child	0.032	0.020	0.174	1.577	0.118
	SCISAS adult	0.060	0.018	0.359	3.252	0.002
Detachment ^b^ (Model 2)	(Constant)	1.009	0.134		7.531	<0.0001
	SCISAS child	0.001	0.021	0.007	0.060	0.952
	SCISAS adult	0.052	0.019	0.324	2.721	0.008
Antagonism ^c^ (Model 3)	(Constant)	0.610	0.122		4.996	<0.0001
	SCISAS child	–0.007	0.019	–0.046	–0.375	0.708
	SCISAS adult	0.033	0.017	0.234	1.901	0.060
Disinhibition ^d^ (Model 4)	(Constant)	1.071	0.123		8.735	<0.0001
	SCISAS child	0.015	0.019	0.093	0.761	0.449
	SCISAS adult	0.026	0.017	0.180	1.476	0.143
Psychoticism ^e^ (Model 5)	(Constant)	0.772	0.143		5.401	<0.0001
	SCISAS child	0.025	0.023	0.130	1.103	0.273
	SCISAS adult	0.045	0.020	0.262	2.229	0.028

^a^ R = 0.483; F = 14.906; *p* < 0.0001; ^b^ R = 0.328; F = 5.907; *p* = 0.004; ^c^ R = 0.210; F = 2.256; *p* = 0.110; ^d^ R = 0.247; F = 3.185; *p* = 0.046; ^e^ R = 0.355; F = 7.086; *p* = 0.001.

## Discussion

To our knowledge, this is the first study to investigate the potential correlation between self-reported separation anxiety in both childhood and adulthood and the personality domains outlined in the DSM-5-dimensional model. The most notable finding of this study is that elevated levels of adult separation anxiety, as measured by the SCI-SAS-A, were positively associated with the domains of Negative Affectivity, Detachment, and Psychoticism. Unexpectedly, separation anxiety during childhood did not show any significant correlation with the evaluated personality domains.

Given that Negative Affectivity, in contrast to Affective Stability, encompasses facets such as Anxiousness and Separation Insecurity, its association with separation anxiety in adulthood is not unexpected. This aligns with the existing literature, which indicates that PID-5 Negative Affectivity, separation anxiety, and intolerance of uncertainty tend to co-occur in non-clinical samples [21]. A recent study on the intergenerational transmission of separation anxiety showed significant associations between adult separation anxiety and internalizing symptoms in both parents and their offspring [22]. A longitudinal study conducted by the same research group on a community sample of 565 women found significant associations between levels of adult separation anxiety and negative emotionality, independent of co-occurring psychopathology, mood-state biases, or overlap with other temperamental or personality traits [23]. Our findings further showed that adult separation anxiety is associated with elevated levels of Detachment, which, along with Negative Affectivity, is a domain included in the proposed criteria for borderline (Cluster B) and avoidant (Cluster C) personality disorders. In this context, focusing on personality domains and traits rather than on traditional categories of personality disorders may represent a step forward in understanding the main dimensional contributors to categorial diagnoses. It is also important to note that patients with anxiety disorders, comorbid SAD and personality disorders showed poor recovery in global functioning [24], a multidimensional construct which has been negatively correlated with the Detachment domain [25]. Separation anxiety may thus be considered both a potential precursor to personality disorders and a modifying factor in illness history, contributing to the deterioration of global functioning in adults. Further studies are required to test this hypothesis from a developmental perspective.

Finally, high levels of adult separation anxiety were positively associated with alterations in the Psychoticism Domain. To date, no data are available in the literature regarding this correlation. This gap in knowledge may be explained by the difficulties of recruiting samples with high levels of Psychoticism using a categorial approach, such as individuals with Schizotypal Personality Disorder. This finding seems more consistent with the disorganized attachment style described in classical attachment theory.

In our study, the level of separation anxiety symptoms in childhood was not correlated with personality domains expressed in adulthood. This result contrasts with the current literature, which emphasizes the role of early SAD in personality development. A brief report [17] showed that high levels of childhood SAD were associated with higher rates of Cluster B and Cluster C personality disorders, whereas adult SAD alone had less impact on that association. However, it should be underlined that the study sample consisted of 397 patients with anxiety disorders, 33% of whom had panic disorder and agoraphobia. It is worth mentioning that the presence of panic disorder may have influenced the findings, given the well-established shared vulnerability between panic and early separation anxiety. A survey on volunteer parents of individuals with borderline personality disorder examined vulnerability factors across three developmental periods: infancy and toddlerhood, childhood, and adolescence. It found that excessive separation anxiety in infants and toddlers (under 5 years old), along with unusual sensitivity and the difficulty in self-soothing, were the main developmental precursors of borderline personality disorder in males [26]. By adolescence, the profile was dominated by acting out, impulsivity, and aggressive and self-destructive behaviors; however, separation anxiety at subsequent developmental stages has not yet been investigated.

Our study is the first to highlight the possibility that adult separation anxiety levels are significantly associated with personality domains. It has been hypothesized that the personal and social demands arising during the developmental stage of emerging adulthood may trigger vulnerability to separation anxiety and emotional lability [27], as individuals transition from dependency on a caregiver to independence as adult members of society. Although most individuals navigate this transition successfully, our results suggest that separation anxiety, alongside other risk factors, should be considered a potential developmental obstacle to achieving a rich and adult personality organization. Beyond the developmental transition, adulthood today is marked by experiences of social separation and isolation, with mental health consequences that have yet to be fully explored. The recent COVID pandemic highlighted the vulnerability of us all to these experiences with a global rise in mental illness [28]. Further research into separation anxiety may help us to understand some of the mechanisms driving this increase.

Several limitations should be considered. The primary limitations are the cross-sectional design, and the small sample size, which may affect the generalizability of the results. Moreover, the use of self-report instruments could have been influenced by individual factors, such as defensiveness, social desirability, and the “halo effect”, which refers to the lack of discrimination among behaviors. Demographic factors, which might have influenced the result, were not included in the regression analysis. Furthermore, the brief version of the PID did not allow for an analysis of the contribution of each facet to the specific domains. Despite these limitations, our study provides additional support for the hypothesis that when a vulnerability to separation anxiety emerges during adulthood, it can lead to the expression of maladaptive personality traits, potentially impacting socio-relational functioning and quality of life. This seems to be a useful approach for further research, aimed at identifying disorder antecedents and factors involved in personality development beyond traumatic experiences.

## Conclusions

The results of this study indicate that adult SAD is associated with alterations in specific personality domains. These findings highlight the importance for health professionals to assess adult SAD in clinical settings, especially in the context of personality disorders. Our findings should encourage further research to clarify the association between personality and separation anxiety, a transversal construct intertwined with various psychiatric disorders. This association influences clinical expression, outcomes, and recovery, potentially representing a target for specific interventions.

## Availability of Data and Materials

The datasets used and/or analyzed during the current study are available from the corresponding author on reasonable request.
